# Neutrophils: a key component in ECMO-related acute organ injury

**DOI:** 10.3389/fimmu.2024.1432018

**Published:** 2024-09-13

**Authors:** Mingfu Zhang, Shiping Li, Junjie Ying, Yi Qu

**Affiliations:** ^1^ Department of Pediatrics, Key Laboratory of Birth Defects and Related Diseases of Women and Children (Ministry of Education), West China Second University Hospital, Sichuan University, Chengdu, China; ^2^ Key Laboratory of Chronobiology (National Health Commission), West China Second University Hospital, Sichuan University, Chengdu, China

**Keywords:** extracorporeal membrane oxygenation (ECMO), neutrophils, neutrophil extracellular traps (NETs), acute organ injury, inflammatory responses

## Abstract

Extracorporeal membrane oxygenation (ECMO), as an extracorporeal life support technique, can save the lives of reversible critically ill patients when conventional treatments fail. However, ECMO-related acute organ injury is a common complication that increases the risk of death in critically ill patients, including acute kidney injury, acute brain injury, acute lung injury, and so on. In ECMO supported patients, an increasing number of studies have shown that activation of the inflammatory response plays an important role in the development of acute organ injury. Cross-cascade activation of the complement system, the contact system, and the coagulation system, as well as the mechanical forces of the circuitry are very important pathophysiological mechanisms, likely leading to neutrophil activation and the production of neutrophil extracellular traps (NETs). NETs may have the potential to cause organ damage, generating interest in their study as potential therapeutic targets for ECMO-related acute organ injury. Therefore, this article comprehensively summarized the mechanism of neutrophils activation and NETs formation following ECMO treatment and their actions on acute organ injury.

## 1 Introduction

Extracorporeal membrane oxygenation (ECMO) is an extracorporeal life support technique which can maintain tissue oxygenation for days to weeks to save the lives of reversible critically ill patients with life threatening respiratory or/and cardiac failure when conventional treatments fail. Prior to this, we were more familiar with cardiopulmonary bypass, which facilitates open heart surgery for a number of hours (1, 2). With advancements in technology and increased clinical indications, ECMO is increasingly being widely used in the treatment of critically ill patients. However, the potential complications associated with ECMO cannot be ignored. These complications include coagulation abnormalities, bleeding, infections, and acute organ injury, all of which can significantly increase the mortality rate of patients (3–6). Acute organ injury encompasses conditions such as acute kidney injury, acute brain injury, and acute lung injury, among others (7, 8). Ongoing research is focused on exploring the underlying pathophysiological mechanisms of these complications. It is noteworthy that the activation of neutrophils and the production of neutrophil extracellular traps (NETs) are likely to play crucial roles in ECMO-related acute organ injury (9, 10). Neutrophils are vital for innate immune defense, and the study of NETs has garnered considerable attention in the field of neutrophil biology. NETs not only provide protective functions against microbial invasion but also contribute to the development of sterile inflammation, hemostasis activation and autoimmune diseases (11, 12). Therefore, it is essential to recognize that during extracorporeal circulation, the interaction between blood and circuit surfaces, as well as the influence of mechanical forces, neutrophils can undergo activation and trigger the production of NETs through a series of cascading and interrelated reactions, likely contributing to the occurrence of ECMO-related acute organ injury (13, 14). Hence, targeting neutrophils and NETs may emerge as a potential therapeutic strategy for preventing ECMO-related acute organ injury. Currently, targeted drugs for NETs mainly focus on inhibiting their formation and promoting their degradation (15). However, the mechanisms of action and clinical feasibility of drugs targeting NETs are still in need of further investigation and validation before they can be widely implemented in clinical practice. In this review, we aim to comprehensively summarize the mechanism of neutrophils activation and NETs formation following ECMO treatment, and their potential role in the pathophysiology of developing organ dysfunction on ECMO and could be a novel and exciting new pathway for treatment.

## 2 Extracorporeal membrane oxygenation

**Figure 1:**
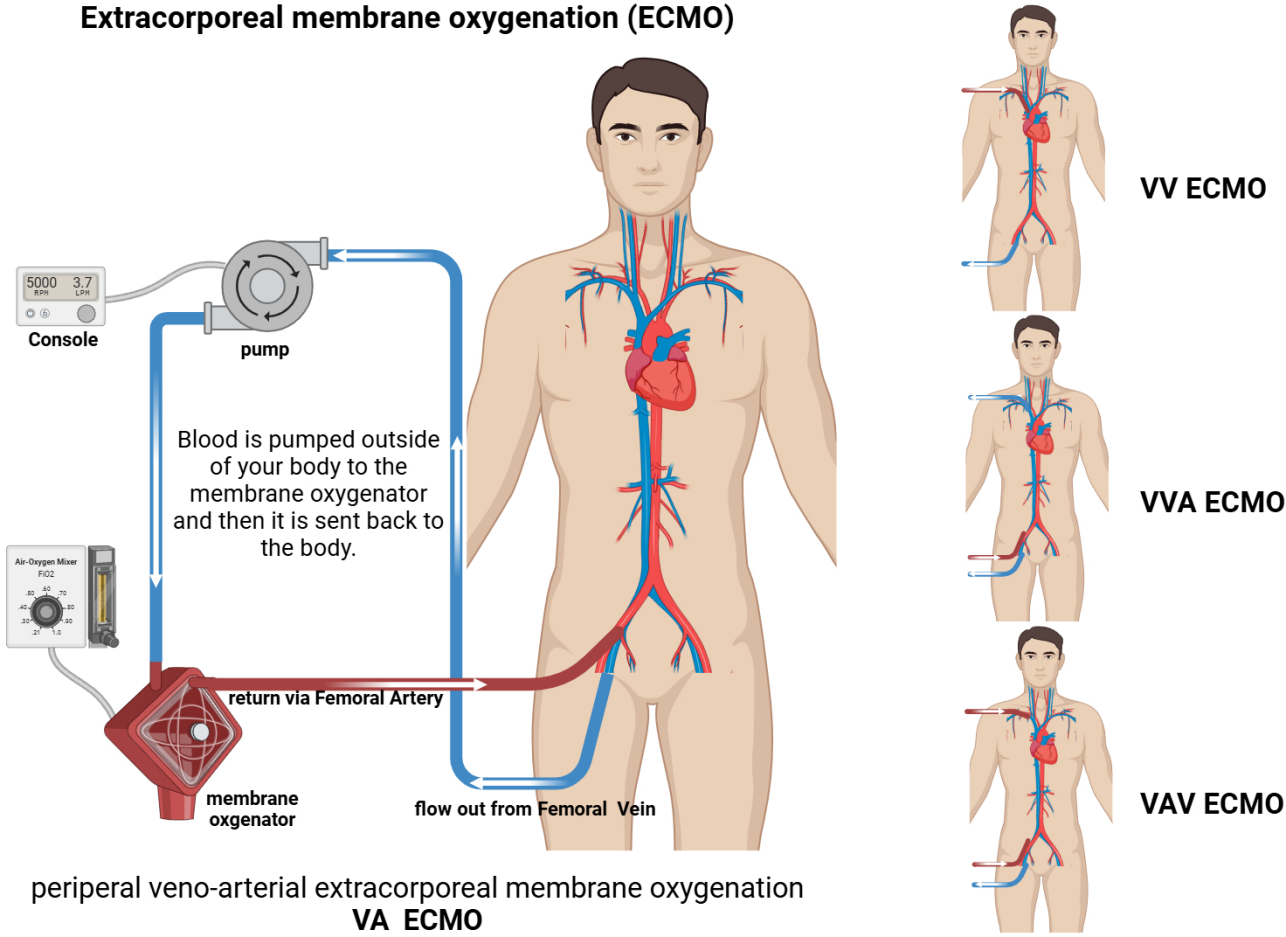
Schematic of extracorporeal membrane oxygenation (ECMO). VA ECMO: drainage Femoral Vein; inflow Femoral Artery. VV ECMO: drainage Femoral Vein; inflow Jugular Vein. VVA ECMO: drainage Jugular Vein and Femora Vein; inflow Femoral Artery. VAV ECMO: drainage Femoral Vein; inflow Femoral Artery and Jugular Vein.

ECMO is an extracorporeal life support technique for critically ill patients that provides respiratory and/or cardiac circulatory support to successfully reverse the disease process and ensure survival (1, 16). The basic principle of ECMO is that venous blood flows out of the patient’s body through an outflow tube under the action of a centrifugal/rolling pump, passes through an oxygenator to simulate pulmonary ventilation, and the oxygenated blood is then returned to the patient’s body through an inflow tube (17). Depending on the clinical indication(s), veno-arterial ECMO (VA-ECMO) and veno-veno-ECMO (VV-ECMO) are available. It is important to note that the use of ECMO should be preceded by a comprehensive assessment of the individual patient’s condition, the most basic factor of which is that the disease must be reversible (18, 19). Multiple placement models, such as veno-veno-arterial ECMO (VVA-ECMO) and veno-arterial-veno ECMO (VAV-ECMO) have emerged in circuit designs (20). According to the existing literature, the former is mainly used in specific patient populations in which VA-ECMO treatment is not able to completely drain and significantly improve hypoxic symptoms, adding an additional drainage tube, thus improving drainage and reducing load. An additional venous cannula is also added when the size of the vessel precludes adequate drainage with one venous cannula according to a neonatal case report (21). The latter combines the advantages of VV-ECMO and VA-ECMO, providing both respiratory and circulatory support, which is highly beneficial for critically ill patients with combined heart and respiratory failure (20, 22) (**Figure 1**). It is important to emphasize that there have been few clinical trials investigating triple tube intubation; therefore, further clinical research evidence is needed to support and guide clinical practice.

Despite advances in technology, however, ECMO-related complications remain a significant cause of increased mortality in patients, such as those experiencing neurological injury (23), acute kidney injury (AKI) (24), bleeding (3), and thrombotic events (25), any of which may be fatal for those who are critically ill. One study reported a median incidence of acute neurological complications of 13% (1%–78%) (26). Moreover, 60%–74% of neonates and children undergoing ECMO experience AKI (7). Blood-related complications and their incidence in critically ill children treated with ECMO include bleeding (43.7%), thrombosis (27.6%), and hemolysis (34.3%) (4).

An increasing number of recent studies have focused on ECMO-related complications. These can be broadly classified into two categories: ECMO device-related (oxygenators, circuit cannulas, ventilators); and patient pathophysiological reactions (thrombosis, bleeding, hemolysis, infection). Contact of the patient’s blood with the non-endothelial surfaces is equivalent to contact with an exogenous foreign body, which triggers the activation of the body’s rejection mechanism and a series of other reactions, such as the induction of an immune inflammatory response, oxidative stress, and activation of the coagulation system, as well as changes in body cells including vascular endothelial cells, leukocytes, and platelets. Alterations in pathophysiological responses are important causes of ECMO-related complications.

## 3 Neutrophils and NETs

Neutrophils, a type of granulocyte, are derived from granulocyte/macrophage precursors in the bone marrow and are produced at a high rate but have a short survival period, accounting for approximately 60%–70% of human peripheral blood leukocytes (27). They are the main cells participating in the acute inflammatory response and are rapidly recruited to the locus of pathogenic infections to destroy the pathogen through multiple mechanisms (28). In recent years, the understanding of neutrophils has no longer been limited to infectious diseases, and their role in conditions, such as tumors, autoimmune diseases, and sterile tissue inflammation, is becoming better understood (11, 12, 29).

ECMO-related complications include a high incidence of acute organ damage. One of the pathophysiological mechanisms involved in acute tissue injury is the activation of the systemic inflammatory response due to the contact of blood with non-endothelial surfaces, leading to the release of various inflammatory and coagulation factors and, ultimately, to the activation of effector cells (13, 30). The most important of these is the activation of neutrophils, which are the main players in ECMO-related acute organ injury (9, 10, 31, 32). Activated neutrophils reach and infiltrate into tissues and organs through the blood circulation, leading to acute organ injury.

Next, we describe the process of neutrophil activation and its contribution to acute organ injury with a further understanding of the pathophysiological mechanisms of acute organ injury in ECMO.

## 4 Mechanisms mediating neutrophil activation and NETs formation in ECMO

**Figure 2:**
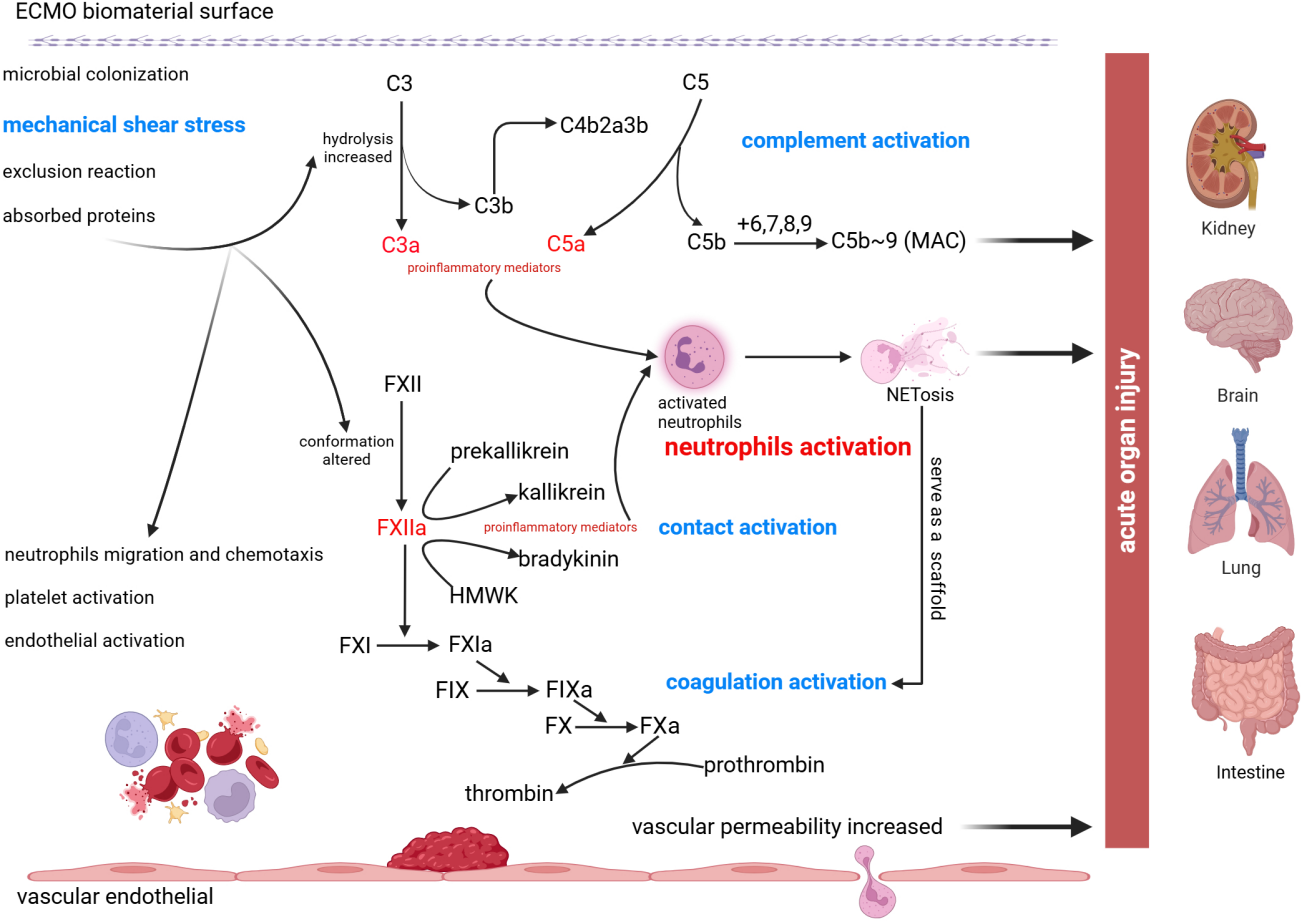
Schematic of neutrophil activation and NETs formation following ECMO treatment. Due to the contact of blood with abiotic materials and mechanical shear stress, C3 hydrolysis increased and FXII conformation altered which promote the production of proinflammatory mediators. Cross-cascade activation of the complement system, the contact system, and the coagulation system causes neutrophils activation and formation of NETs, ultimately leading to acute organ injury, including acute kidney injury, acute brain injury, acute lung injury, and acute intestinal injury.

An increasing number of studies have shown that activation of the inflammatory response plays an important role in the development of acute organ injury, and very important pathophysiological mechanisms are likely secondary to cross-cascade activation of the complement, contact, and coagulation systems, as well as the mechanical forces of the circuitry, which lead to neutrophil activation and the production of NETs (**Figure 2**).

### 4.1 The complement system

The complement system is an important component of innate immunity in humans. Among the 3 initiation pathways of complement activation, the classical pathway (CP) is induced by the formation of antigen-antibody complexes, the mannose-binding lectin pathway is activated by mannose residues on the surface of the pathogen, and the bypass (alternative) pathway is activated by polysaccharides on the surface of the pathogen. According to previous studies, the alternative complement pathway is instantly activated upon initiation of ECMO and is one of the earliest defense responses mounted by the host (33).

Several studies have demonstrated elevated levels of complement activators during ECMO. Vallhonrat et al. measured the levels of complement activation in 2 adult patients with acute respiratory distress syndrome requiring ECMO treatment, and found that early activation of the complement system may be responsible for the initiation of the inflammatory response (34). Graulich et al. found that after initiation of ECMO in neonates, the complement system was rapidly activated due to blood contact with the circuit surface and not the underlying disease (35). In addition, with improvements in circuit design and pump technology, studies have identified methods to reduce complement activation, such as the use of centrifugal pumps and circuit-bound heparin (36, 37). Although these findings were previously based on the cardiopulmonary bypass (CPB) model, the principles of ECMO are essentially the same.

It has been shown that the extracorporeal circulation line is subject to microbial colonization (38, 39) as well as increased C3 hydrolysis due to contact of its own blood with non-endothelial surface biomaterials (13). Thus, in extracorporeal circulation, the complement activation pathway is mainly the alternative complement pathway (40), which is responsible for the production of the proinflammatory allergenic toxins C3a and C5a and the membrane attack complex (MAC), and regulates the activation of neutrophils (41). At the same time, the alternative pathway of complement activation exhibits positive feedback amplification effects and there is cross-promotion among the 3 pathways, resulting in comprehensive activation of the complement system. Among these, the intermediate product C5a is a potent and effective pro-inflammatory factor (42), with biological effects including activation of neutrophils, promotion of neutrophil migration and chemotaxis, enhancement of neutrophil adhesion to the vascular endothelium, and allergenic toxin effects (leading to vascular smooth muscle contraction and increased vascular permeability) (43–45). Activation of the complement system is an important cause of neutrophil activation in ECMO circulation.

### 4.2 The contact system

Components of the contact system include coagulation factors XII and XI, high-molecular-weight kininogen (HMWK), and prokinetic peptide releasing enzyme (46, 47). Some studies have confirmed that the contact system has no role in normal hemostasis and that patients deficient in contact factors prokinetic kininogen, HMWK, and coagulation factor XII(FXII) do not bleed (48). When blood comes into contact with the non-endothelial surface of the circuity, the conformation of FXII is altered, and a small amount of activated FXII(FXIIa) is produced, with circulating levels of FXIIa reaching a maximum within 10 mins of ECMO initiation (49). The cleaved product, FXIIa, initiates the coagulation system and promotes the simultaneous conversion of the prokinetic peptide-releasing enzyme to active kinase and high-molecular-weight kininogen to bradykinin. In addition, FXIIa can activate the complement protein C1 (50, 51), an active component in the classical complement activation pathway. Thus, FXIIa plays a crucial role in complement activation. Blocking excessive activation of this factor is a key target for effectively inhibiting the activation of the contact system, and the use of novel inhibitory antibodies to neutralize FXIIa has been shown to reduce the inflammatory response in animal models of ECMO, as well as thrombosis prevention (52, 53).

Upon activation of the contact system, kinases and bradykinins drive inflammation and promote coagulation (54). The release of kinases during ECMO activates the endogenous coagulation system and directly activates neutrophils (55, 56). Bradykinin acts as an inflammatory mediator that leads to vasodilation and increases the vascular permeability and chemotaxis of neutrophils (57, 58). The effect of bradykinin during ECMO has not been extensively studied; however, in CPB-related studies, bradykinin levels have, to a large extent, been shown to be elevated (59), probably because CPB bypasses the lung tissue (where bradykinin is inactivated). Based on this principle, it can be hypothesized that bradykinin has a more pronounced effect on VA-ECMO than on VV-ECMO. Similarly, in ex situ organ perfusion (ESOP) (60), the absence of the biological role of organs, such as the liver, leads to the inability to scavenge oxygen radicals generated by oxidative stress, thus making such a technique more challenging compared with physiological conditions (30).

### 4.3 Endogenous and exogenous coagulation systems

Under normal physiological conditions, coagulation mechanisms include both coagulation and anticoagulation, and the dynamic balance between them plays a key role in maintaining normal blood flow and preventing coagulation (61). Maintenance of this dynamic balance is mainly dependent on 3 elements: the structural integrity of the vascular wall and regulatory function of the vascular endothelium; the speed and flow of blood; and the quantity and quality of platelets and coagulation factors involved in coagulation. During ECMO, significant non-physiological changes occur in the above 3 elements, including: direct contact of the patient’s blood with the non-endothelial cell surface of the circuity; high shear forces; turbulent flow of blood under the influence of pump equipment; and dilution, adsorption, activation, and consumption of blood components, which have an important impact on the dynamic balance of the coagulation mechanism. Thus, contact between the patient’s blood and the extracorporeal circulation surface during ECMO activates the systemic coagulation system. It noteworthy that ECMO related non-physiological changes do not include the physiologic changes that could go along with a disease process such as sepsis, including disseminated intravascular coagulopathy (DIC) or hepatic injury. After initiation of the coagulation process, thrombus formation also requires the involvement of thro mbin (62), which requires 2 necessary conditions for sustained production: initiation of the coagulation process; and aggregation of activated coagulation factors into a procoagulant phospholipid surface (48). As mentioned above, initiation of the coagulation process is responsible for the production of FXIIa, which activates the intrinsic coagulation pathway, leading to thrombin formation. Regarding the formation of procoagulant phospholipid surfaces, there have been hypotheses suggesting the adsorption of proteins from the serum to the surface of the circuit material and further adsorption of leukocytes and platelets; however, the exact underlying mechanism of action between blood and biomaterials is not well understood. Clinical studies have shown that coagulation activation during ECMO initially leads to the deposition of fibrin, platelets, and leukocytes on the circuit surface (63), which can develop into sarcoid thrombosis in some patients (64). The incidence of thrombosis within the ECMO circuit and membrane oxygenator (MO) has been reported to be 3%–22% and 21%, respectively (65). The deposition of fibrin and formation of thrombi cause fibrin chains to intertwine with the thrombus, resulting in increased resistance to blood flow. In this case, pump speed is increased accordingly, which in turn increases mechanical stress and causes cellular damage as well as further activation of the coagulation hierarchy. In pediatric patients undergoing ECMO, the increased risk for thrombosis is related to the underlying disease and the artificial ECMO circuit itself (56). During the new coronavirus pneumonia epidemic, up to 20% of critically ill patients experienced thromboembolic events during ECMO treatment, which increased the risk for organ failure and death (66–68).

Coagulation depletion is also associated with ECMO treatment. In a pilot study investigating VA-ECMO in sheep, coagulation factors, platelet counts, and antithrombin levels decreased at ECMO onset, but with a significant increase in neutrophil levels (69). Clinical trials have also compared *in vivo* coagulation between the VV and VA modalities, reporting that those undergoing VA-ECMO exhibited similar coagulation-related markers of consumptive blood disorders and relatively less use of heparin; however, the 2 did not exhibit statistically significant differences in thrombosis and bleeding events (70). Thus, hematological alterations associated with ECMO treatment are multifactorial. This finding suggests the need for additional clinical studies to confirm individualized anticoagulation treatment strategies in the context of ECMO.

The ECMO-associated inflammatory response can be mitigated by inhibiting excessive activation of the contact system; however, conventional anticoagulation therapy (e.g., sodium citrate and heparin) has no inhibitory effect on the contact system, only on downstream coagulation factors. FXI and FXII inhibitors have been tested in *in vitro* studies (71, 72). EP-7041, a small molecule FXI antagonist, has demonstrated safety and good tolerability in Phase 1 human studies (73). In addition, antisense oligonucleotides are used to inhibit FXI biosynthesis, and the mechanism of action is that these oligonucleotides reduce FXI levels by specifically targeting binding to FXI messenger RNA and causing its degradation, thereby reducing thrombus formation (74, 75). Inhibitors targeting the active site of FXII have been shown to inhibit thrombus formation in extracorporeal membrane oxygenators in animal ECMO models (76). A clinical trial evaluated the effectiveness and safety of garadacimab, a factor XII inhibitor, for the prevention of hereditary angioedema. One study offers a crucial initial insight into alterations in the contact pathway in children receiving ECMO support. FXI and FXII antigen and function change during ECMO (77). However, there are currently no clinical studies on inhibitors of factors XII and XI in the treatment of patients on ECMO. What’s more, Bivalirudin, a direct thrombin inhibitor without need for Antithrombin III (ATIII) and without binding to Platelet factor 4(PF4). Many centers are starting to use Bivalirudin as primary anticoagulation in ECMO (78). Some studies have shown that Bivalirudin seemed to be more efficacious and safer than unfractionated heparin(UFH) for patients undergoing ECMO and a significant reduction in the transfusion requirements of composite blood products in pediatric patients on ECMO (79, 80). These new anticoagulant therapies are associated with fewer bleeding events than traditional anticoagulant drugs and the effects of contact system activation could be suppressed by direct inhibition of FXII, FXI and thrombin. Combined with the mechanism of contact system activation of coagulation and inflammatory response, these anticoagulant therapies may also have the potential to reduce the incidence of thromboembolic events as well as neutrophil activation and NETs formation in patients undergoing ECMO.

### 4.4 Activation of neutrophils following ECMO treatment

As previously described, the activation of the inflammatory response, coagulation cascade system, and endothelial cells in patients treated with ECMO is intertwined and complex; however, the specific mechanisms of action remain poorly understood. In summary of available studies, the exogenous contact of blood with non-endothelial surfaces leads to the production of FXIIa by the coagulation system, which promotes the production of pro-inflammatory cytokines and has a direct effect on leukocytes, platelets, and vascular endothelial cells.

In an *in vitro* circulatory model, the activation of neutrophils is still mainly due to the activation of the complement system, which can significantly reduce neutrophil levels if C5 antagonists are administered. In the presence of chemotactic and adhesion molecules produced by endothelial cells, neutrophils infiltrate and accumulate in organs, leading to the development of acute organ inflammation, which is believed to be an important cause of acute organ damage associated with ECMO.

Neutrophils in the circuit after ECMO initiation rapidly adhere to the MO or pulmonary vascular epithelium, and marginalization causes a transient decrease in neutrophil levels (81); however, these neutrophils upregulate the expression of adhesion proteins and enhance the cytotoxic response (82). In addition, it has been shown that neutrophils are altered early in VA-ECMO by elevated plasma levels of granulocyte colony stimulating factor, leading to an increase in the proportion of immature neutrophils, which exert immunosuppressive effects, reducing phagocytic activity and pathogen clearance, and is an important cause of acquired infections in critically ill patients treated with extracorporeal circulatory support (83).

### 4.5 Mechanical shear stress exposure

The basis of neutrophil function is the transition from a quiescent state to an activated state, known as depolarization, which can be induced by chemical signaling or mechanical disturbances (84). Thus, sustained mechanical deformation of neutrophils can induce depolarization.

Neutrophil activity and function can be altered in the ECMO circuit owing to pump characteristics. Blood circulates through the circuit and is affected by mechanical shear stress. The higher the rotational speed, the higher the activation level of neutrophils and the higher the phagocytosis capacity (85). However, neutrophil activation levels and phagocytic capacity gradually decrease. In addition, the magnitude of the shear force affects the activation status of neutrophils, and an excessively large shear force can lead to a loss of neutrophil activity and function (14). In conclusion, during the initial stages of ECMO, neutrophils are rapidly activated by the mechanical forces of the circuitry, which can aggravate acute organ injury.

### 4.6 NETs

NETs are extracellular networks of DNA, histones, microbicidal proteins, and oxidative enzymes that are released by activated neutrophils in response to various stimuli, including viral and inflammatory cytokines (86, 87). Although NETs are believed to have antimicrobial functions in innate immunity (88), their dysregulation can trigger and propagate inflammation and thrombosis (28, 89), leading to severe tissue damage.

A study using a sheep model suggested that NETs may be involved in ECMO-associated thrombosis and play an important role in the inflammatory response, serving as a target and early warning marker for intervention in thrombosis (90). It was reported that the activation of the complement system leads to the activation of neutrophils and that the release of NETs serves as a mediator of the activation of the coagulation system, with complex interaction(s) between them (89, 91). This suggests that NETs play an important role in inducing the activation of the inflammatory response and coagulation cascade, which in turn may be a very important pathophysiological mechanism in ECMO.

## 5 Neutrophil and NETs action on ECMO-associated acute organ injury

In recent years, many studies have focused on the complications of acute organ injury in critically ill patients treated with ECMO, which usually include acute kidney, brain, lung, and intestinal injuries. The incidence of acute organ injuries is high, which seriously affects the success rate of ECMO treatment and can even lead to death.

### 5.1 AKI

AKI is a common complication in patients treated with ECMO, and its incidence varies widely across studies due to individual patient differences and different clinical scenarios. Among neonates, 60%–74% undergoing ECMO have AKI and, in a cohort study of adults treated with ECMO (7, 92), the estimated incidence of AKI and severe AKI requiring renal replacement therapy (RRT) was 62.8% and 44.9% (93), respectively. It usually occurs 24–48 h after the start of ECMO and increases the risk for death in critically ill patients (7). However, studies have shown that patients treated with ECMO combined with RRT do not exhibit a significant reduction in mortality (93).

NETs formation is not only a simple host immune defense mechanism but also drives pathophysiological conditions associated with sterile inflammation and autoimmunity, leading to various AKI and chronic kidney injury. NETs formation plays an important role in the pathogenesis and disease progression of kidney injury (94–96). Studies investigating ischemic kidney injury have shown that deletion of the alternative complement pathway attenuates injury from ischemia-reperfusion (97), as well as in C3a knockout mice, reduces the formation of NETs after ischemia, thereby protecting the kidney (96). Combined with our previously described evidence, we hypothesized that in ECMO, complement system activation and NETs production after neutrophil activation may be important pathological mechanisms contributing to AKI.

NETs have been observed to be associated with various causes of AKI, including glomerulonephritis, ischemic AKI, septic AKI, and Systemic lupus erythematosus (SLE) (94, 98–100). However, it is surprising that existing studies investigating interventions targeting NETs formation have produced inconsistent results. For example, research conducted by J.S. Knight et al. suggests that the use of PAD4 inhibitors can reduce NETs formation and subsequently decrease organ damage (101). Conversely, studies by Gordon RA et al. indicate that while inhibiting NETs formation is possible, it does not alleviate the extent of kidney injury (102). Nevertheless, in animal models of ischemic AKI, the involvement of PAD4 in NETs formation leads to post-ischemic kidney damage, suggesting a potential therapeutic significance of PAD4 inhibitors in post-ischemic kidney injury (103). Thus, based on the aforementioned potential pathological mechanisms, it is hypothesized that inhibiting NETs formation or promoting NETs degradation could potentially prevent the occurrence of ECMO-related acute kidney injury in the early stages. However, it is crucial to approach these potential treatment strategies with caution and seek further research support, as the responsiveness to treatment may vary among different patients.

### 5.2 Acute brain injury

ECMO-associated brain injuries―both acute and chronic―are not uncommon in patients undergoing such treatment (8). Acute brain injury significantly reduces patient survival and usually includes intracranial hemorrhage and ischemic brain injury (104, 105). Simultaneously, it has been shown that in children who survive discharge from hospital after undergoing ECMO, follow-up observations reveal many problems with growth and neuropsychological development (106, 107).

Neutrophils activated in a series of cascades release inflammatory factors that increase the permeability of the cerebral vascular epithelial layer (31, 32). The formation of NETs promotes the activation of the coagulation system, and thrombi are formed in the extracorporeal circulation or dislodge with the flow of blood to block the cerebral vasculature (108, 109). Neutrophil activation produces inflammatory mediators that induce adaptive immune responses. Extracorporeal circulation enhances the inflammatory phenotype of adaptive immune cells, and the inflammatory mediators produced by the adaptive immune response can have toxic effects on the developing central nervous system in children (110).

Existing research has confirmed the pathological mechanisms of NETs in ischemic stroke and traumatic brain injury, focusing on the role of PAD4 in exacerbating brain damage through NETs formation, as well as the potential for improving neural injury through DNase I-mediated NETs degradation (111, 112). This suggests that both PAD4 and DNase I hold promise as potential therapeutic targets. In a study conducted by Mu Q et al., it was demonstrated that an elevated neutrophil count in clinical patients positively correlates with the severity of brain injury. The study primarily investigated a delivery system loaded with PAD4 and effectively validated its ability to prevent NETs formation at the site of brain injury, thereby exerting neuroprotective effects in animal experiments (113). Therefore, if clinical research confirms the involvement of NETs in ECMO-related acute brain injury, it would become a critical target for preventing complications associated with brain damage.

### 5.3 Acute lung injury

Due to the presence of extensive capillary beds in the lungs and the abundance of immune cells in the lung parenchyma, the systemic inflammatory response activated by ECMO renders lung tissue extremely susceptible to acute injury (114, 115). During this process, the inflammatory response leads to increased pulmonary vascular permeability, thereby exacerbating accumulation of pulmonary extravascular fluid.

Acute respiratory distress syndrome (ARDS) is characterized by severe inflammation and damage to the lung air-blood barrier, resulting in significant and often irreversible impairment of respiratory function (116). Pneumonia and sepsis are the most common triggers for ARDS (117). In recent years, ECMO has emerged as the standard of care in specialized ICUs for patients with severe ARDS (118, 119). The primary pathophysiology underlying ARDS involves extensive inflammation and disruption of the lung barrier (117). Activated resident alveolar macrophages (AM) and alveolar type II epithelial cells (AEC II) release cytokines and chemokines (120), recruiting immune cells from circulation to the lungs, including a substantial number of neutrophils (121). Excessive activated neutrophils migrate and infiltrate the interstitium and the formation of NETs contributes to lung injury (116). It is important to note that a small-scale clinical trial indicated that blood markers of neutrophil extracellular traps (NETs) do not show an increase in patients with COVID-19-related ARDS following the initiation of ECMO. Therefore, further clinical data are needed to validate this observation. In addition, a delayed sustained inflammatory response has been observed days after implementation of VA-ECMO, of which the underlying mechanism may be the presence of low circulating endotoxin concentrations that sustain complement activation and cytokine release (122, 123). A DNase I delivery system that mimics the inflammatory chemotactic effect of neutrophils has been shown to effectively prevent inflammatory damage to lung tissues in an LPS-induced acute lung injury mouse model (124), suggesting that targeting NETs to alleviate acute lung inflammation is also a feasible approach.

### 5.4 Acute intestinal injury

Relevant experimental animal studies have shown that ECMO is an independent risk factor for intestinal barrier dysfunction, which leads to bacterial translocation (125). Intestinal villi are damaged due to apoptosis of epithelial cells (126), and intestinal mast cells may act as a reservoir of inflammatory factors, activating the release of large quantities of inflammatory factors after ECMO initiation (127). The initiation of intestinal inflammation can induce the aggregation of neutrophils, which, upon activation, generate NETs. Excessive NETs formation is linked to the pathology of inflammatory intestinal diseases (128). Importantly, several studies have highlighted the role of NETs in the development of intestinal injuries during sepsis (129), and heightened NETosis may lead to dysfunction in intestinal microcirculation (130). The formation of NETs may also be a significant contributing factor in ECMO-related acute intestinal injury.

## 6 NETs-targeted therapy: possibilities and limitations

Current clinical treatments for ECMO-related acute organ injury, primarily focus on symptomatic support, as well as anti-inflammatory and anticoagulants. More targeted approaches are needed that can effectively reduce the formation of NETs and enhance their clearance (**Table 1**). Dimethyl fumarate (DMF) has been shown to inhibit the activation of neutrophils and the formation of NETs (137). Furthermore, clinical studies have demonstrated significant therapeutic effects of DMF in patients with psoriasis and multiple sclerosis (131, 132). It was found that DMF has an effect on peripheral blood mononuclear cells, modulating NETs formation (137), which supports the beneficial effects of DMF under inflammatory conditions.

**Table 1:** Clinical and preclinical trials involving possible NETs-targeted drugs.

Drug	Study design	Phase	Disease	Outcomes	Refs
Dimethyl fumarate	Multicentre, randomised, double-blind	2	MS	Neuroprotective and anti-inflammatory	(131)
	Randomized, double-blind	3	MS	Improve clinical outcome Neuroprotective and immunomodulatory	(132)
Metformin	Randomized, open-label, non-blind	2	SLE	Decreases in clinical flares, prednisone exposure, and body weight Decrease NETs formation	(133)
Tofacitinib	Randomized double-blind	1	SLE	Significant decreases in levels of circulating NETs	(134)
PAD4 inhibitors	Preclinical	/	Ischemia/reperfusion-induced AKI	Block NETs formation Improve the clinical course of inflammatory diseases in experimental mice	(103)
DNase I	Preclinical	/	Traumatic brain injury	Degrade NETs Attenuate inflammation in experimental mice	(113)
	Randomized, open label, parallel assignment	2	COVID-19	Significant reduction in inflammation	(135)
nNIF	Preclinical	/	Sepsis	Decrease NETs formation and inflammatory cytokine levels Improve survival	(136)

NETs, neutrophil extracellular traps; MS, multiple sclerosis; SLE, Systemic Lupus Erythematosus; AKI, acute kidney injury; PAD4, peptidyl arginine deiminase 4; DNase I, Deoxyribonuclease I; nNIF, neonatal NET-inhibitory factor.

In addition, metformin has been found to improve impaired NETs clearance. Metformin, an AMPK activator, has been shown to enhance the phagocytic capacity of macrophages derived from ARDS patients, resulting in increased uptake of apoptotic neutrophils and NETs (138). A proof-of-concept trial investigating metformin as an add-on treatment for mild or moderate SLE demonstrated that metformin down-regulated the NETs mtDNA-PDC-IFNα pathway, leading to clinical improvement of SLE and reduced its reliance on prednisolone (133). However, the direct evidence regarding metformin’s impact on reducing NETs formation was not provided in this study. Nevertheless, the available evidence suggests that metformin holds promise as a potential therapeutic option for inflammatory diseases characterized by excessive accumulation of NETs due to neutrophil activation. Further research is warranted in multicenter, randomized, placebo-controlled trials to investigate its potential in this regard.

Besides, tofacitinib, a JAK inhibitor, has been found to regulate both the formation and degradation of NETs. Treatment with Tofacitinib had no effect on peripheral blood neutrophil count but demonstrated the ability to reduce spontaneous or LPS-induced NETosis (139). In a randomized trial involving SLE patients, significant reduction in NET complexes was observed in individuals with the STAT4 risk allele who received Tofacitinib treatment, while no impact was observed in STAT4 risk allele-negative patients or those in the placebo group (134). These findings suggest that the response to Tofacitinib treatment may vary among patients with different genetic backgrounds. Therefore, further experimental validation is necessary to determine its potential application in other inflammatory diseases.

Furthermore, DNases I and PAD4 inhibitors, which are key players in inhibiting NETs, have exhibited anti-inflammatory effects in conditions such as NETs-related kidney injury and acute respiratory distress syndrome (140, 141). DNases I could degrade NETs DNA backbone, and several clinical trials showed protective effects of rhDNase1 in COVID-19 patients, which could reduce inflammation (135, 142). Dornase alfa is recombinant human DNase 1. A case series reveals that five mechanically ventilated patients with COVID-19—three of whom required VV-ECMO—were successfully extubated and discharged from the hospital following the initiation of dornase alfa administration (143). This result is promising and underscores the potential significance of DNases I. PAD4 inhibitors could block NET formation via inhibition of histone citrullination. In an animal model of ischemic acute kidney injury, PAD4 played a role in the formation of NETs, which contributed to renal damage after ischemia and the use of PAD4 inhibitors may have therapeutic implications for the treatment of ischemic AKI (103). Moreover, a delivery system loaded with PAD4 has been effectively validated to prevent the formation of NETs at the site of brain injury, thereby exerting neuroprotective effects (113).

A fascinating discovery by Yost et al. revealed the presence of a unique NET inhibitor called neonatal NET-inhibitory factor (nNIF) in umbilical cord blood (144). They also unveiled a series of nNIF-related peptides (NRPs) that possess the ability to selectively hinder NET formation without compromising other crucial neutrophil antimicrobial functions or platelet responses (144). In a preclinical investigation, nNIF treatment demonstrated comparable efficacy in managing neonatal sepsis when compared with established anti-NET strategies. Notably, it significantly enhanced survival rates in a neonatal mouse model of sepsis when combined with sub-optimal meropenem treatment (136).

Further research is necessary to investigate the effectiveness of these treatments in ECMO-related acute organ injury. It is crucial to consider the potential risks associated with targeted therapies for neutrophils, such as compromising the host defense system and increasing the risk of infections and even may trigger deleterious effects (15). Thus, a deeper understanding of the molecular mechanisms underlying neutrophil involvement in ECMO-related acute organ injury is essential for identifying biomarkers and targets that can specifically and accurately regulate excessive neutrophil activation and NETs accumulation. This knowledge has the potential to alleviate systemic inflammatory responses and improve the success rate of treatment.

## 7 Discussion

The primary focus of the present article is the activation of the inflammatory response in the context of extracorporeal circulation, in which the main driver is the activation of neutrophils. However, it is noteworthy that, in critically ill patients treated with ECMO, the presence of a primary disease can also contribute to the development of an inflammatory response in the organism system. For example, patients requiring VA-ECMO, who experience severe cardiogenic shock, can also develop a systemic inflammatory response (145). Therefore, it is difficult to determine whether the rise in inflammatory factor levels in the organism is caused by ECMO alone. However, in addition to the pathophysiological development of critical illness caused by the effects of the primary disease, it is undeniable that extracorporeal circulation itself induces an inflammatory response, which further intensifies the systemic inflammatory response and leads to a systemic “storm” of inflammatory factors, further developing into the adverse outcome of multi-organ failure. Furthermore, it is noteworthy that a small-scale clinical trial indicated that blood markers of neutrophil extracellular traps (NETs) do not show an increase in patients with COVID-19-related ARDS following the initiation of ECMO (146). Nonetheless, additional comprehensive clinical research data from larger cohorts is required to substantiate the potential rise of NETs in clinical patients. The main mechanism of action for the inflammatory response is likely secondary to the contact of blood with abiotic surfaces in the extracorporeal circulation, thus activating a series of reactions, the most direct effect of which is the activation of complement, contact, and coagulation systems. This cascade leads to the activation of neutrophils and formation of NETs, which are important pathological factors involved in the acute inflammatory response, likely contributing to the organ damage seen in patients supported on ECMO. Further studies evaluating the role of neutrophils and NETs in patients on ECMO are required and may lead to a novel approach to therapeutic targets to prevent additional organ damage.

While we have previously discussed potential treatment approaches, it is important to note that the response to the modulation of neutrophil activity can vary among different patients. This variation is dependent on factors such as the specific disease type and severity of the patient. For example, patients with autoimmune diseases may experience symptom alleviation through interventions targeting neutrophil activity regulation. However, patients who are currently infected or have a compromised immune system may face an elevated risk of infection with such interventions. In any scenario, personalized treatment strategies and meticulous monitoring are indispensable to ensure the best possible therapeutic outcomes.

## 8 Conclusion

Extracorporeal membrane oxygenation (ECMO) is considered the ultimate support for critically ill patients, but it is crucial to address the complications associated with ECMO-related acute organ injury as they significantly impact treatment success. Excessive activation of neutrophils and the subsequent accumulation of NETs have the potential to be a main factor in the development of ECMO-related acute organ injury, which is amplified by interconnected cascading reaction systems. Therefore, it holds immense theoretical significance and clinical potential to identify the primary targets involved in this process and develop targeted drugs to enhance treatment success in critically ill patients.
